# Translation initiation with exotic amino acids using EF-P-responsive artificial initiator tRNA

**DOI:** 10.1093/nar/gkad496

**Published:** 2023-06-19

**Authors:** Takayuki Katoh, Hiroaki Suga

**Affiliations:** Department of Chemistry, Graduate School of Science, The University of Tokyo, 7-3-1 Hongo, Bunkyo-ku, Tokyo 113-0033, Japan; Department of Chemistry, Graduate School of Science, The University of Tokyo, 7-3-1 Hongo, Bunkyo-ku, Tokyo 113-0033, Japan

## Abstract

Translation initiation using noncanonical initiator substrates with poor peptidyl donor activities, such as *N*-acetyl-l-proline (AcPro), induces the N-terminal drop-off-reinitiation event. Thereby, the initiator tRNA drops-off from the ribosome and the translation reinitiates from the second amino acid to yield a truncated peptide lacking the N-terminal initiator substrate. In order to suppress this event for the synthesis of full-length peptides, here we have devised a chimeric initiator tRNA, referred to as tRNA^iniP^, whose D-arm comprises a recognition motif for EF-P, an elongation factor that accelerates peptide bond formation. We have shown that the use of tRNA^iniP^ and EF-P enhances the incorporation of not only AcPro but also d-amino, β-amino and γ-amino acids at the N-terminus. By optimizing the translation conditions, e.g. concentrations of translation factors, codon sequence and Shine-Dalgarno sequence, we could achieve complete suppression of the N-terminal drop-off-reinitiation for the exotic amino acids and enhance the expression level of full-length peptide up to 1000-fold compared with the use of the ordinary translation conditions.

## Graphical Abstract

**Graphical Abstract:**
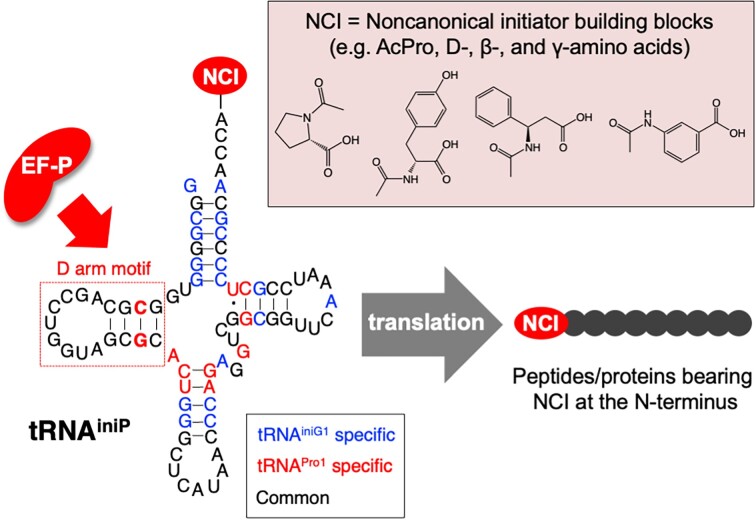

## INTRODUCTION

Peptides bearing noncanonical N-terminal building blocks, such as proline (Pro), d-amino, β-amino and γ-amino acids, are an attractive platform for development of novel bioactive peptides. For instance, introduction of N-terminal Pro contributes to stabilization of turn and helical conformations of peptides (1,2). d-Amino, β-amino and γ-amino acids also exhibit unique and strong folding propensities, such as turn/helix inducing abilities. Thus, peptides containing these amino acids can be folded into various structures with more drug-like properties (3–12). We can expect high binding affinity and inhibitory activity against target molecules, improved membrane permeability and proteolytic stability for these foldamer peptides (13–17). These noncanonical substrates can be ribosomally incorporated into peptides by genetic code reprogramming. However, clean expression of such exotic peptides introducing noncanonical substrates is a long-standing challenge in this field because their incorporation efficiencies are generally low (18–23).

In canonical translation initiation in prokaryotes, P-site *N*-formylmethionyl-tRNA^ini^ (fMet-tRNA^ini^) is recognized by one of the initiation factors, IF3, to survey the stability of codon-anticodon interaction and thereby relocated to the active position (24–26). If IF3 fails in the relocation, fMet-tRNA^ini^ eventually drops-off from ribosome and translation reinitiates from the second aminoacyl-tRNA at the A site to give a truncated peptide lacking the N-terminal fMet, which is called a reinitiated peptide (RiP) (27). This event is referred to as the N-terminal drop-off-reinitiation. Incorporation of noncanonical substrates with poor peptidyl donor activity, such as *N*-acetyl-l-proline (AcPro), causes more frequent drop-off-reinitiation (27,28). Therefore, it is far more difficult to introduce such exotic amino acids at the N-terminus.

**Figure 1.**
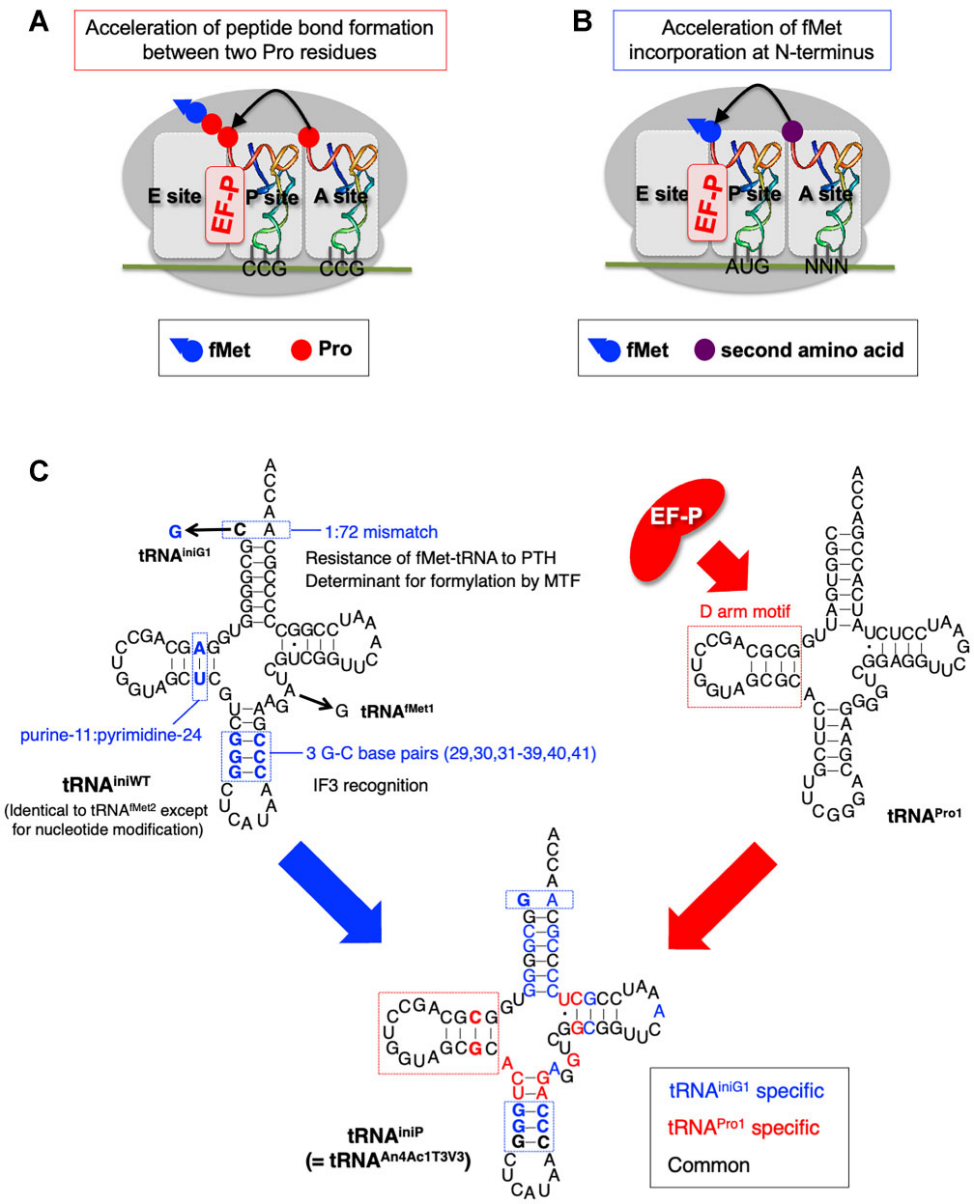
EF-P recognizes the D-arm of P-site tRNA for acceleration of peptide bond formation. (**A**) EF-P-mediated acceleration of peptide bond formation between two consecutive Pro residues at P and A site in translation elongation (30,31). EF-P recognizes the specific D-arm motif of the P-site peptidyl-prolyl-tRNA^Pro^ for the acceleration (35). (**B**) EF-P mediated acceleration of peptide bond formation between P-site fMet-tRNA and A-site amino acid (32,33). This event occurs at translation initiation. (**C**) Development of a novel initiator tRNA that can be efficiently recognized by EF-P for acceleration of peptide bond formation. The nucleotide sequence of tRNA^iniWT^ is identical to that of *E. coli* tRNA^fMet2^ except for the nucleotide modifications due to *in vitro* transcription of tRNA^iniWT^. The 5'-end C is replaced by G for tRNA^iniG1^. The D-arm motif of tRNA^Pro1^ can be recognized by EF-P (indicated by red dotted line). By combining the structural features of tRNA^iniWT^ and tRNA^Pro1^, the engineered tRNA, tRNA^iniP^, was devised. The nucleotides derived from tRNA^iniG1^ are indicated by blue, those from tRNA^Pro1^ by red, and the common ones in black. Note that nucleotide modifications are omitted in this figure.

The drop-off-reinitiation event also occurs in translation elongation when introducing consecutive Pro residues into nascent peptides (29). The low peptidyl donor/acceptor abilities of Pro cause ribosome stalling, where EF-G triggers mistranslocation of P-site peptidyl-Pro-tRNA and A-site Pro-tRNA toward E and P site, respectively. Then, translation reinitiates from the P-site Pro-tRNA to give a truncated peptide lacking the N-terminal region. However, it is known that the specific translation factor, EF-P, accelerates peptide bond formation between the consecutive Pro residues, thereby alleviating the ribosomal stalling (Figure 1A) (30,31). We have reported that EF-P can also prevent the drop-off-reinitiation event in elongation induced by consecutive Pro (29). Since EF-P functions not only in elongation but also in initiation (32–34)(Figure 1B), here we hypothesized that the N-terminal drop-off-reinitiation can also be alleviated by EF-P. If this is the case, we can efficiently synthesize such exotic peptides containing AcPro at the N-terminus in the presence of EF-P. However, in the preceding reports, only fMet was evaluated as the N-terminal substrate; Noncanonical, less reactive peptidyl donor substrates, such as AcPro, have not been thoroughly examined for EF-P-assisted enhancement of peptide bond formation (32,33). Therefore, we first aimed at confirming that EF-P is able to enhance peptide bond formation between the N-terminal AcPro and the second amino acid, thereby alleviating N-terminal drop-off-reinitiation.

We previously reported that EF-P recognizes the specific D-arm structure of tRNA^Pro^ isoacceptors, tRNA^Pro1^, tRNA^Pro2^ and tRNA^Pro3^, for acceleration of peptidyl transfer of Pro (35). Therefore, if the D-arm of tRNA^ini^ is substituted with those of tRNA^Pro^ isoacceptors, peptidyl transfer between the N-terminal AcPro and the second amino acid could be further enhanced. Here we aim at developing such an engineered tRNA^ini^ variant by merging the structural features of wild-type tRNA^ini^ and tRNA^Pro^ isoacceptors for more efficient incorporation of AcPro at the N-terminus (Figure 1C). This novel initiator tRNA was referred to as tRNA^iniP^. Since we have also reported that IF3, EF-G and RRF are involved in suppression of the N-terminal drop-off-reinitiation (27), the concentrations of these translation factors are also optimized for AcPro incorporation. Consequently, the use of tRNA^iniP^ charged with AcPro as well as other exotic nonproteinogenic amino acids, e.g. d-amino, β-amino and γ-amino acids, under the optimized conditions for the protein factors enabled us to efficiently express the full-length peptides including such amino acids at the N-terminus. We also demonstrated macrocyclization of peptides by introducing N-terminal *N*-chloroacetylated amino acids, which reacted with a sulfhydryl group of downstream Cys to form a thioether bond.

## MATERIALS AND METHODS

### Preparation of tRNA^ini^ variants and flexizymes

Template DNAs for tRNA^ini^ variants and flexizymes (dFx and eFx) were prepared by extension of forward and reverse extension primer pairs, and PCR using forward and reverse PCR primer pairs (See Supplementary Table S1 for details). Transcription of tRNA^ini^ and flexizymes was conducted at 37°C for overnight in 250 μl and 2 ml, respectively, of the following reaction mixtures: 40 mM Tris–HCl (pH 8.0), 22.5 mM MgCl_2_, 1 mM dithiothreitol (DTT), 1 mM spermidine, 0.01% Triton X-100, 3.75 or 5 mM nucleoside triphosphate (NTP) mix, 5 or 0 mM guanosine monophosphate (GMP), 0.04 U/μl RNasin RNase inhibitor (Promega) and 0.12 μM T7 RNA polymerase. 200-μl- or 2-ml-scale PCR products were added to the above reaction mixture for transcription of tRNA^ini^ and flexizymes, respectively. The concentrations of NTP mix were 3.75 mM for tRNA^ini^ and 5 mM for flexizymes, and those of GMP were 5 and 0 mM, respectively. The transcribed tRNA^ini^ and flexizymes were treated with RQ1 DNase (Promega) at 37°C for 30 min and purified on 8% (tRNA^ini^) or 12% (flexizymes) polyacrylamide gels containing 6 M urea. The resulting RNAs were eluted from the gel, precipitated by ethanol and dissolved in water.

### Preparation of aminoacyl-tRNAs

Aminoacylation of tRNA^ini^ variants was carried out at 0°C in a following reaction mixture: 50 mM HEPES–KOH (pH 7.5), Bicine-KOH (pH 9.0) or CHES–KOH (pH 10.0), 600 mM MgCl_2_, 20% DMSO, 25 μM dFx or eFx, 25 μM tRNA^ini^ variants and 5 mM activated amino acids. l-proline-3,5-dinitrobenzyl ester (Pro-DBE), *N*-acetyl-d-tyrosine-cyanomethyl ester (Ac^D^Tyr-CME), *N*-acetyl-d-tryptophane-cyanomethyl ester (Ac^D^Trp-CME), l-β-homophenylglycine-3,5-dinitrobenzyl ester (^β^Phg-DBE) and 3-aminobenzoic acid-cyanomethyl ester (^3^Abz-CME) were utilized as the activated amino acids for charging Pro, Ac^D^Tyr, Ac^D^Trp, ^β^Phg and ^3^Abz, respectively. The reaction was conducted for 2, 3, 3, 22 and 144 h, respectively. Acylations of ^β^Phg and ^3^Abz were performed at pH 9.0 and 10.0, respectively, and the other substrates were acylated at pH 7.5. These activated amino acids were synthesized by previously reported methods (36–38). dFx was used for DBEs and eFx for CMEs. The resulting aminoacyl-tRNAs were precipitated by ethanol, washed twice with 70% ethanol containing 0.1 M sodium acetate (pH 5.2), and dissolved in 1 mM sodium acetate (pH 5.2). For *N*-acetylation of Pro-tRNA^ini^, ^β^Phg-tRNA^ini^ and ^3^Abz-tRNA^ini^, 250 pmol aminoacyl-tRNA^ini^ was dissolved in 60 μl 0.3 M sodium acetate/0.5 M acetic anhydride solution (pH 5.2), incubated for 30 min at 25°C, and then recovered by ethanol precipitation. For *N*-chloroacetylation of Pro-tRNA^ini^ and ^3^Abz-tRNA^ini^, 500 pmol aminoacyl-tRNA^ini^ was dissolved in 75 μl 0.3 M sodium acetate/40 mM chloroacetic anhydride solution (pH 5.2), incubated for 5 min at 25°C and recovered by ethanol precipitation. The resulting *N*-acetyl- or *N*-chloroacetyl-aminoacyl-tRNAs were washed twice with 70% ethanol containing 0.1 M sodium acetate (pH 5.2) and dissolved in 1 mM sodium acetate (pH 5.2).

### Translation of model peptides

The model peptide P1 was translated using template DNAs that encode mRNAs mR1 − mR6. The template DNAs were prepared by extension of forward and reverse extension primer pairs, and PCR using forward and reverse PCR primer pairs (see Supplementary Table S1 for details). Translation was carried out at 37°C for 30 min in a 2.5 μl-scale FIT system of the following composition unless otherwise designated: 50 mM HEPES–KOH (pH 7.6), 100 mM potassium acetate, 12.6 mM magnesium acetate, 2 mM ATP, 2 mM GTP, 1 mM CTP, 1 mM UTP, 20 mM creatine phosphate, 2 mM spermidine, 1 mM DTT, 1.5 mg/ml *Escherichia coli* total tRNA, 1.2 μM *E. coli* ribosome, 0.6 μM methionyl-tRNA formyltransferase, 2.7 μM IF1, 3 μM IF2, 1.5 μM IF3, 0.1 μM EF-G, 20 μM EF-Tu/Ts, 0 or 5 μM EF-P, 0.25 μM RF2, 0.17 μM RF3, 0.5 μM RRF, 4 μg/ml creatine kinase, 3 μg/ml myokinase, 0.1 μM inorganic pyrophosphatase, 0.1 μM nucleotide diphosphate kinase, 0.1 μM T7 RNA polymerase, 0.13 μM AspRS, 0.11 μM LysRS, 0.02 μM TyrRS, 0.5 mM Asp, 0.5 mM U-^13^C:U-^15^N-Lys, 0.5 mM Tyr, 20 μM aminoacyl-tRNA^ini^ variants, 0.5 μM DNA template and 0.5 μM internal control peptides (control-P1-FLP and control-P1-RiP). The sequences of control-P1-FLP and control-P1-RiP are identical to those of translated-P1-FLP and translated-P1-RiP, respectively, except for the U-^13^C:U-^15^N-Lys labeling. In titration of IF3, EF-G, EF-P, RRF and aminoacyl-tRNA^ini^, their concentrations were changed accordingly as indicated in Figure 4, 5 and Supplementary Figure S2. Note that the internal control peptides were not added in the experiments shown in Figure 5, where U-^13^C:U-^15^N-Lys was utilized for the translation in the presence of EF-P and unlabeled Lys for that in the absence of EF-P.

### MALDI-TOF mass spectrometry of model peptides

Translated peptides were desalted with SPE C-tip (Nikkyo Technos) and cocrystallized with α-cyano-4-hydroxycinnamic acid on a sample plate. For the analysis shown in Figure 5, two translation solutions derived from EF-P(+) and EF-P(−) experiments were mixed together, and then subjected to C-tip and cocrystallization. MALDI-TOF mass spectrometry (MS) was performed by UltrafleXtreme (Bruker Daltonics) in reflector-positive mode. A peptide calibration standard II (Bruker Daltonics) was used for external mass calibration.

## RESULTS

### Ribosomal incorporation of *N*-acetyl-l-proline at the N-terminus

**Figure 2.**
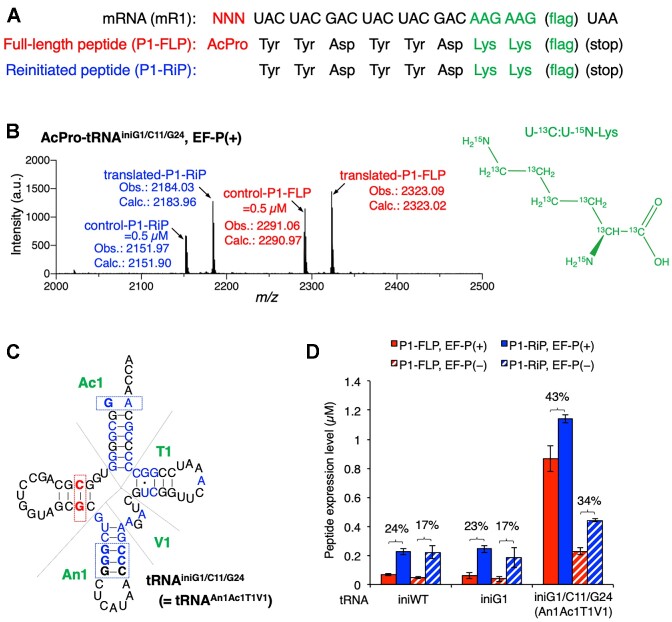
Translation of a model peptide for evaluation of tRNA^ini^ variants in AcPro incorporation at the N-terminus. (**A**) Sequences of mRNA, mR1 and the corresponding peptide sequence, P1. P1-FLP is a full-length peptide bearing AcPro at the N-terminus. P1-RiP is a reinitiated peptide lacking AcPro. U-^13^C:U-^15^N-Lys (monoisotopic mass: 154.12) was introduced into the translated P1-FLP and P1-RiP instead of unlabeled Lys (monoisotopic mass: 146.11), and indicated by green. The mRNA and peptide sequences for flag are GAC-UAC-AAG-GAC-GAC-GAC-GAC-AAG and Asp-Tyr-Lys-Asp-Asp-Asp-Asp-Lys, respectively. (**B**) MALDI-TOF MS of translation products. Translation was carried out using tRNA^iniG1/C11/G24^ in the presence of 5 μM EF-P. Calculated (calc.) and observed (obs.) *m/z* values of [M + H]^+^ are indicated. Concentrations of the translated P1-FLP and P1-RiP were determined by their relative peak intensities to those of the internal control P1-FLP and P1-RiP (0.5 μM). (**C**) Secondary structure of tRNA^iniG1/C11/G24^. The anticodon stem, acceptor stem, T-stem and variable loop of this tRNA are referred to as An1, Ac1, T1 and V1, respectively. This tRNA is also called tRNA^An1Ac1T1V1^. (**D**) Quantification of the expression levels of P1-FLP and P1-RiP. Translation of these peptides was conducted using tRNA^iniWT^, tRNA^iniG1^ and tRNA^An1Ac1T1V1^ in the presence and absence of EF-P. Numbers above the bars indicate P1-FLP%. n = 3. Error bars, S.D.

To observe N-terminal drop-off-reinitiation, AcPro was introduced into a model peptide P1 using an mRNA mR1 (Figure 2A). For incorporation of AcPro, we first tested three initiator tRNA variants, tRNA^iniWT^, tRNA^iniG1^ and tRNA^iniG1/C11/G24^ (Figure 1C for tRNA^iniWT^ and tRNA^iniG1^, 2C for tRNA^iniG1/C11/G24^). All of these tRNAs are derived from *E. coli* tRNA^fMet2^ but lack nucleotide modifications due to *in vitro* transcription. In addition, tRNA^iniG1^ and tRNA^iniG1/C11/G24^ have G1 and G1/C11/G24 mutations, respectively. G1 mutation aimed at improving transcription efficiency and C11/G24 mutation for efficient recognition by EF-P. Since EF-P recognizes D-arm of tRNA^Pro1^ and the only difference in the D-arm of tRNA^fMet2^ and tRNA^Pro1^ is found at this position (Figure 1C, A11/U24 for tRNA^fMet2^ and C11/G24 for tRNA^Pro1^) (35), we assumed that C11/G24 mutation would enhance its recognition by EF-P. Pro was precharged on these tRNAs using dFx, one of flexizyme variants (39), and *N*-acetylated by acetic anhydride to prepare AcPro-tRNA.

Translation was conducted in an *E. coli* reconstituted translation system, referred to as the flexible *in vitro* translation (FIT) system (40), containing U-^13^C:U-^15^N-Lys instead of unlabeled Lys. Thus, the translated peptides were labeled with four U-^13^C:U-^15^N-Lys (Figure 2A, B, indicated by green). Consequently, both full-length P1 (P1-FLP) and reinitiated peptide lacking the N-terminal AcPro (P1-RiP) were detected by MALDI-TOF MS (Figure 2B, a representative result for tRNA^iniG1/C11/G24^ in the presence of EF-P). The expression levels of the translated P1-FLP and P1-RiP were estimated by their relative peak intensities to those of 0.5 μM synthetic internal control peptides, control-P1-FLP and control-P1-RiP, bearing unlabeled Lys. We assumed that the translated and control P1 peptides have equal ionization efficiencies due to the identical amino acid sequences except for the isotope labeling. Consequently, the levels of P1-FLP using tRNA^iniWT^, tRNA^iniG1^ and tRNA^iniG1/C11/G24^ were 0.067, 0.061 and 0.87 μM, respectively, in the presence of EF-P; while 0.045, 0.038 and 0.23 μM, respectively, in the absence of EF-P (Figure 2D). The percentages of P1-FLP [P1-FLP%: P1-FLP / (P1-FLP + P1-RiP) × 100] were 24%, 23% and 43%, respectively, in the presence of EF-P and 17%, 17% and 34%, respectively, in the absence of EF-P (Figure 2D). Both P1-FLP level and P1-FLP% were enhanced by EF-P for all of these tRNAs, indicating that their D-arms could be recognized by EF-P. In addition, tRNA^iniG1/C11/G24^ exhibited 14-fold higher expression level of P1-FLP and 20% higher P1-FLP% compared to the use of tRNA^iniG1^ in the presence of EF-P (0.87 μM versus 0.061 μM and 43% versus 23%), showing that the introduction of the C11/G24 mutation contributed to enhancement of the P1-FLP level and P1-FLP%. Since the enhancement effects were observed even in the absence of EF-P for tRNA^iniG1/C11/G24^, it is likely that its conformational change induced by the C11/G24 mutation is preferable for AcPro incorporation. On the other hand, the G1 mutation did not affect the function of initiator tRNAs. Therefore, we decided to introduce G1 and C11/G24 mutations to all initiator tRNAs hereafter.

### Screening of tRNA^ini^ variants for efficient expression of the full-length peptide

**Figure 3.**
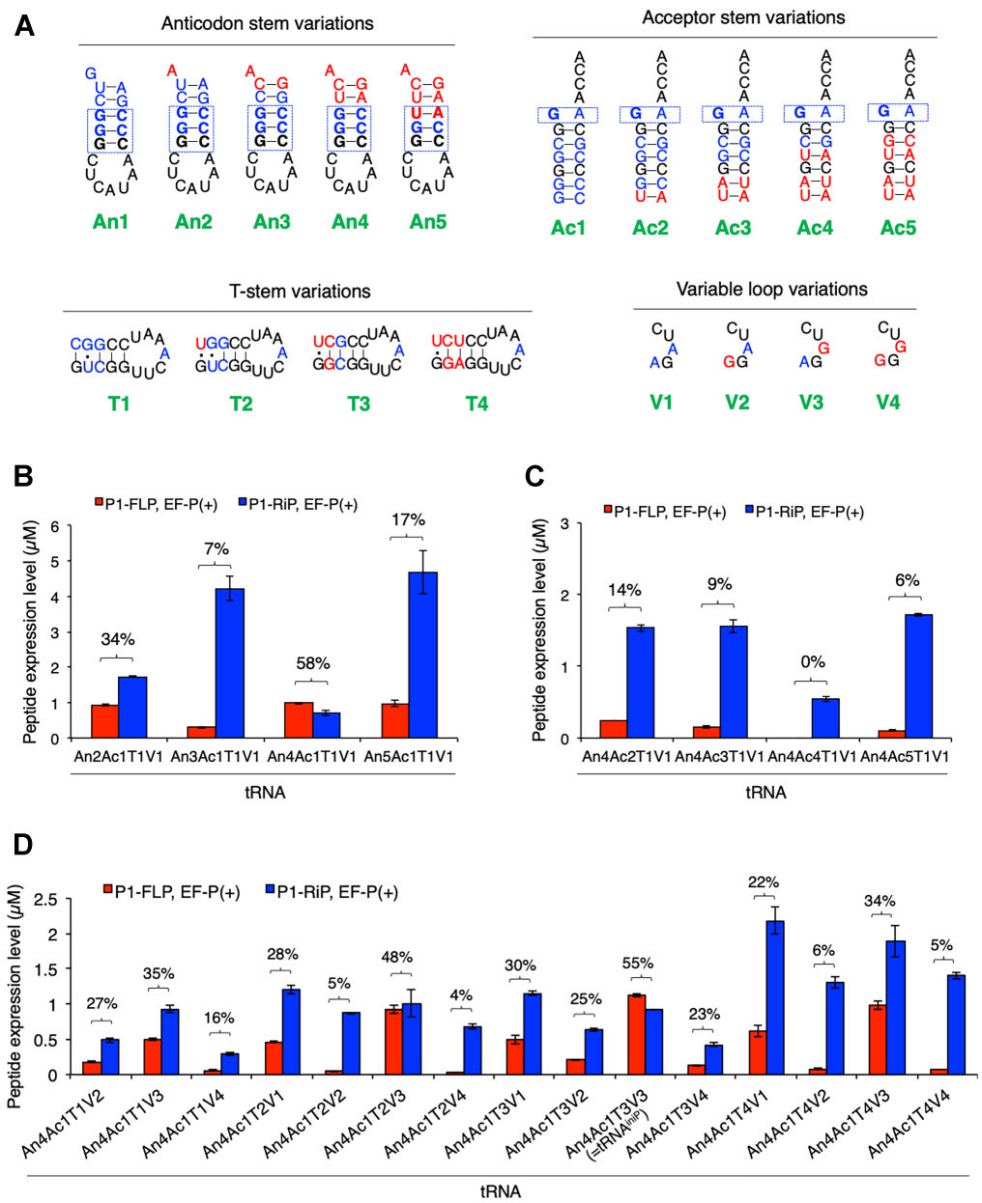
Evaluation of tRNA^ini^ variants bearing mutations at anticodon stem, acceptor stem, T-stem, and variable loop. (**A**) Structural variations of anticodon stem, acceptor stem, T-stem and variable loop introduced into tRNA^ini^. (B–D) Quantification of the expression levels of P1-FLP and P1-RiP for anticodon stem variants (**B**), acceptor stem variants (**C**) and T-stem/variable loop variants (**D**). Translation of the peptides was conducted in the presence and absence of EF-P. Numbers above the bars indicate P1-FLP%. *n* = 3. Error bars, S.D. See also Supplementary Figure S1 for the secondary structures of tRNA^ini^ variants.

The above result motivated us to further optimize the local structures of tRNA^iniG1/C11/G24^ to improve the efficiency of AcPro incorporation into full-length peptides. P1-FLP level and P1-FLP% in the presence of EF-P were used as benchmarks for evaluation of tRNA^ini^ variants. We screened such a tRNA^ini^ variant that shows the highest P1-FLP level and P1-FLP% so that we can efficiently and cleanly express full-length P1-FLP bearing AcPro at the N-terminus using the tRNA^ini^ variant. Here we introduced five anticodon stem variations (An1−5), five acceptor stem variations (Ac1−5), four T-stem variations (T1−4), four variable loop variations (V1−4), and their combinations, where the sequence of tRNA^Pro1^ was partially implanted into tRNA^iniG1/C11/G24^ (Figure 3, Supplementary Figure S1, bases derived from tRNA^iniG1^ are indicated in blue, those from tRNA^Pro1^ in red, and common bases in black). Note that tRNA^iniG1/C11/G24^ has An1, Ac1, T1 and V1 and is referred to as tRNA^An1Ac1T1V1^ hereafter (Figure 2C); other mutants are also named similarly after their local structures: tRNA^AnXAcXTXVX^, where X indicates the numbering of local structures shown in Figure 3A. First, we evaluated anticodon stem variants (Figure 3B, Supplementary Figure S1A, tRNA^An2−5Ac1T1V1^). Among them, tRNA^An4Ac1T1V1^ showed the highest P1-FLP level and P1-FLP% in the presence of EF-P (Figure 3B, 0.98 μM and 58%). Thus, we chose An4 as the best anticodon stem structure. Second, we evaluated acceptor stem variants, where anticodon stem was fixed to An4 (Figure 3C, Supplementary Figure 1B, tRNA^An4Ac2−5T1V1^). Consequently, none of these variants showed higher P1-FLP level nor P1-FLP% than those of tRNA^An4Ac1T1V1^ (Figure 3C, 0−0.24 μM and 0−14%, respectively, in the presence of EF-P). Thus, we decided to keep using Ac1 as the best acceptor stem among Ac1 − 5. Third, combinations of T-stem variations and variable loop variations were evaluated using tRNA^An4Ac1T2−4V2−4^, where anticodon and acceptor stems were fixed to An4 and Ac1, respectively (Figure 3D, Supplementary Figure S1C). For the T-stem variants, T3 showed generally higher P1-FLP level and P1-FLP% than T1, T2 and T4 (Figure 3D). For the variable loop variants, V3 showed higher P1-FLP level and P1-FLP% than V1, V2 and V4 (Figure 3D). Thus, the combination of T3 and V3 resulted in the highest P1-FLP level as well as P1-FLP% (Figure 3D, tRNA^An4Ac1T3V3^, 1.12 μM and 55%, respectively, in the presence of EF-P). Since tRNA^An4Ac1T3V3^ showed the highest P1-FLP level among all tRNAs evaluated in this study, we decided to use this tRNA for further validations. tRNA^An4Ac1T3V3^ is referred to as tRNA^iniP^ hereafter (Figure 1C, Supplementary Figure S1C).

### Optimization of translation conditions for efficient expression of the full-length peptide

**Figure 4.**
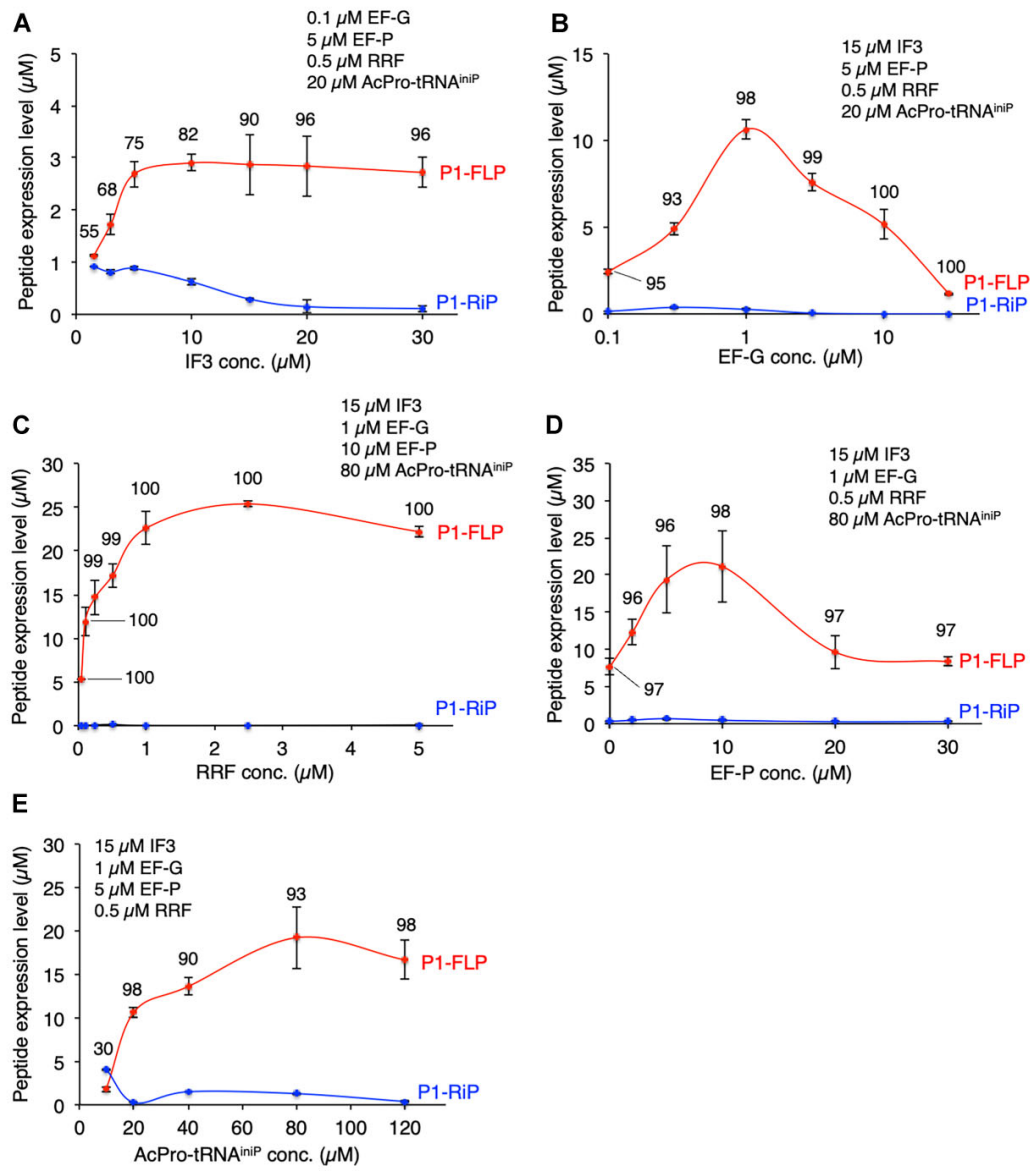
Titration of translation factors and AcPro-tRNA in translation of P1 peptide. (A−E) Titration of IF3 (**A**), EF-G (**B**), RRF (**C**), EF-P (**D**) and AcPro-tRNA (**E**). Red and blue dots indicate the expression levels of P1-FLP and P1-RiP, respectively. Numbers above the red dots indicate P1-FLP%. *n* = 3. Error bars, S.D.

We recently revealed that the N-terminal drop-off-reinitiation is suppressed by IF3, EF-G and RRF (27). Therefore, here we optimized the concentrations of IF3, EF-G and RRF to enhance the P1-FLP level and P1-FLP% (Figure 4A–C). In addition, concentrations of EF-P and AcPro-tRNA^iniP^ were also titrated for the P1-FLP synthesis (Figure 4D, E). In titration of IF3, P1-FLP level plateaued at over 5 μM IF3 and P1-FLP% surpassed 90% at over 15 μM (Figure 4A). Thus, we decided to use 15 μM IF3 for the rest of the experiments. In EF-G titration, P1-FLP level peaked at 1 μM EF-G with 98% P1-FLP% and gradually decreased at the higher EF-G concentrations above 1 μM (Figure 4B), indicating the possibility that too high concentration of EF-G induces frequent mistranslocation and drop-off of AcPro-tRNA^iniP^ from the P site. For RRF and AcPro-tRNA^iniP^ concentrations, P1-FLP level plateaued at over 1 μM RRF and 80 μM AcPro-tRNA^iniP^, respectively (Figure 4C,E). For EF-P concentration, P1-FLP level peaked at 5−10 μM EF-P and gradually decreased at higher EF-P concentrations (Figure 4D). This is likely because such high concentrations of EF-P remain to occupy the ribosomal E site even after the peptidyl transfer completes and thus inhibit the translocation of deacyl-tRNA from P site to E site. We previously observed the same tendencies for elongation of inefficient substrates such as Pro, d-amino-, β-amino-, and γ-amino acids (15,22,23,35,41). Comparing the P1-FLP level at 10 μM EF-P with that at 0 μM EF-P, 2.8-fold improvement was observed (Figure 4D, 21.1 μM and 7.6 μM, respectively), showing that tRNA^iniP^ can be recognized by EF-P for enhancing peptidyl transfer under these reaction conditions. P1-FLP level reached at 25 μM at the optimal translation conditions (Figure 4C, 15 μM IF3, 1 μM EF-G, 2.5 μM RRF, 10 μM EF-P and 80 μM AcPro-tRNA^iniP^), where the N-terminal drop-off-reinitiation event was completely suppressed (P1-FLP% = 100).

Since we recently found that AUG is not necessarily the best initiator codon for incorporation of AcPro but other codons, e.g. AAG, possibly show higher P1-FLP% (27), we tested nine codon-anticodon combinations using tRNA^iniP^ for AcPro incorporation into P1 (Supplementary Figure S2A, codon/anticodon = AUU/AAU, AUU/GAU, AUC/GAU, AUA/UAU, AUG/CAU, AAG/CUU, AGA/UCU, GUA/UAC and GGA/UCC). The anticodon of tRNA^iniP^ was changed accordingly to recognize the corresponding codon sequence (Supplementary Table S1). Note that the concentration of AcPro-tRNA^iniP^ was increased to 160 μM in this analysis. As a result, AUU/GAU, AUC/GAU, AAG/CUU and GUA/UAC showed significantly higher P1-FLP level than the canonical AUG/CAU (Supplementary Figure S2A, 54.8 − 63.4 μM for AUU/GAU, AUC/GAU, AAG/CUU and GUA/UAC, and 25.1 μM for AUG/CAU), though AUU/GAU and AUC/GAU resulted in lower P1-FLP% (89% and 91%, respectively, whereas 99−100% for the other combinations). Thus, we concluded that AAG/CUU and GUA/UAC are preferable codon-anticodon combinations for P1-FLP synthesis.

We also evaluated the effect of Shine-Dalgarno (SD) sequence and the spacer between SD and initiation codon. In addition to mR1, five SD + spacer sequences were evaluated, where AUG and AAG were introduced as initiation codons (Supplementary Figure S2B, mR2−mR6). As a result, we observed a wide range of P1-FLP levels depending on the type of SD + spacer, where mR5 exhibited the highest P1-FLP level for both AUG and AAG codons [Supplementary Figure S2C, 0.39 − 59.1 μM (range of P1-FLP level for all SD + spacer sequences), 43.8 and 59.1 μM for mR5-AUG and mR5-AAG, respectively]. Thus, we concluded that the use of mR5-AAG with 15 μM IF3, 1 μM EF-G, 2.5 μM RRF, 10 μM EF-P and 160 μM AcPro-tRNA^iniP^ would be the best conditions for translation of P1-FLP. Under these conditions, we could obtain 59.1 μM P1-FLP, which is over 1000-fold improvement compared to the use of tRNA^iniWT^ under our conventional translation conditions [Figure 2D, tRNA^iniWT^, EF-P(−), 0.045 μM].

### Ribosomal incorporation of d-amino, β-amino and γ-amino acids at the N-terminus by means of tRNA^iniP^ and EF-P

**Figure 5.**
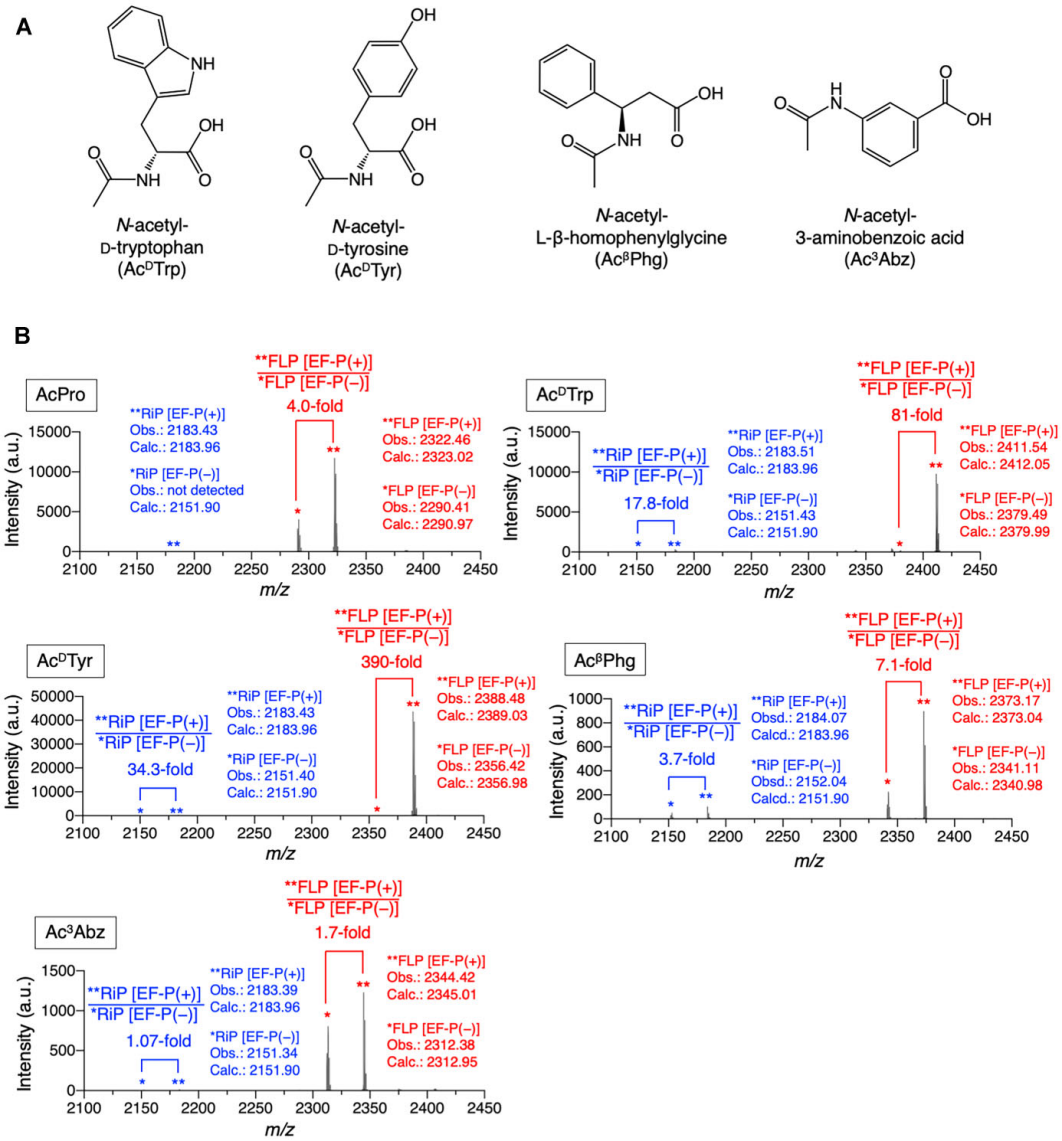
EF-P enhances ribosomal incorporation of d-amino, β-amino and γ-amino acids at the N-terminus in combination with tRNA^iniP^. (**A**) Structures of nonproteinogenic amino acids tested for ribosomal incorporation at the N-terminus. Ac^D^Trp and Ac^D^Tyr are representatives of d-amino acids, while Ac^β^Phg and Ac^3^Abz are representatives of β-amino and γ-amino acids, respectively. (**B**) MALDI-TOF MS of translation products. Translation was carried out using mR5-AAG and aminoacyl-tRNA^iniP^_CUU_ in the presence or absence of EF-P. 15 μM IF3, 1 μM EF-G, 2.5 μM RRF, 10 or 0 μM EF-P and 160 μM aminoacyl-tRNA^iniP^_CUU_ were used. Peptides were labeled with U-^13^C:U-^15^N-Lys for translation in the presence of EF-P, whereas unlabeled Lys was incorporated for the EF-P(−) translation. The EF-P(+) and EF-P(−) translation solutions were mixed together and analyzed by MALDI-TOF MS. Calculated (calc.) and observed (obs.) *m/z* values of [M + H]^+^ are indicated. Fold increases of the P1-FLP level for EF-P(+)/EF-P(−) were estimated by the relative peak intensities.

Next, we aimed at applying tRNA^iniP^ for incorporation of d-amino, β-amino and γ-amino acids using *N*-acetyl-d-tryptophan (Ac^D^Trp), *N*-acetyl-d-tyrosine (Ac^D^Tyr), *N*-acetyl-l-β-homophenylglycine (Ac^β^Phg) and *N*-acetyl-3-aminobenzoic acid (Ac^3^Abz) as their representatives (Figure 5A). These amino acids were precharged on tRNA^iniP^ bearing CUU anticodon, referred to as tRNA^iniP^_CUU_ and introduced at the N-terminus of P1 peptide using mR5-AAG to yield P1-FLP-Ac^D^Trp, P1-FLP-Ac^D^Tyr, P1-FLP-Ac^β^Phg and P1-FLP-Ac^3^Abz. Translation reaction was carried out at 37°C for 30 min in a 2.5-μl reaction solution containing 15 μM IF3, 1 μM EF-G, 2.5 μM RRF, 160 μM aminoacyl-tRNA^iniP^_CUU_ and 0 or 10 μM EF-P. U-^13^C:U-^15^N-Lys was added to the EF-P(+) reaction mix, whereas unlabeled Lys to the EF-P(−) reaction. Then, both EF-P(+) and EF-P(−) reactions were stopped by adding EDTA, mixed together, and applied to MALDI-TOF MS (Figure 5B). To evaluate the enhancement by EF-P, peak intensities of U-^13^C:U-^15^N-Lys-labeled P1-FLP and unlabeled P1-FLP were compared. Consequently, 4.1-fold, 81-fold, 390-fold, 7.1-fold and 1.7-fold improvement of expression levels of P1-FLP-AcPro, P1-FLP-Ac^D^Trp, P1-FLP-Ac^D^Tyr, P1-FLP-Ac^β^Phg and P1-FLP-Ac^3^Abz, respectively, were observed. These results show that tRNA^iniP^ is applicable to EF-P-assisted enhancement of N-terminal incorporation of d-amino, β-amino and γ-amino acids as well as AcPro incorporation.

**Figure 6.**
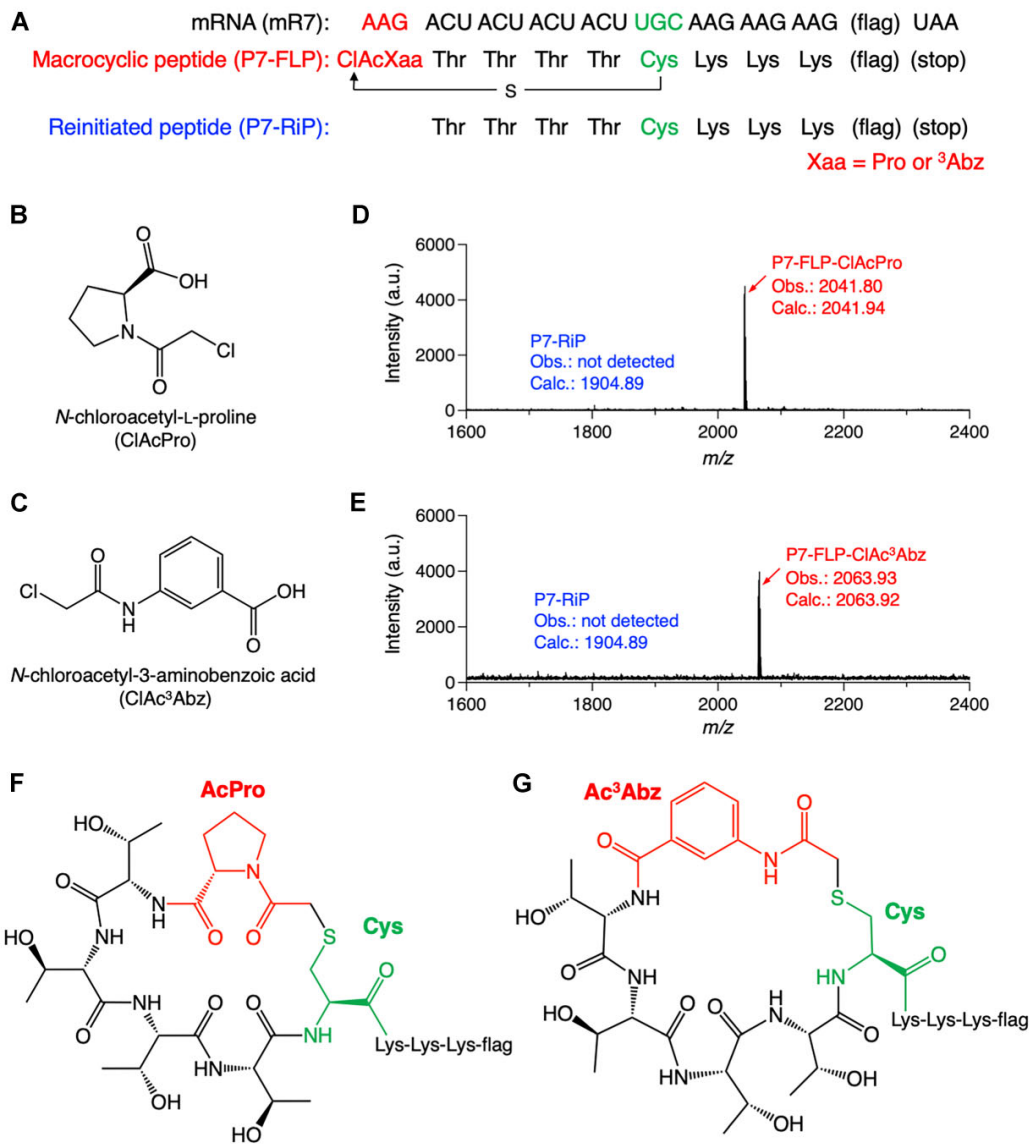
Ribosomal incorporation of *N*-chloroacetyl-l-proline and *N*-chloroacetyl-3-aminobenzoic acid at the N-terminus for macrocyclization of peptides. (**A**) Sequences of mRNA, mR7, and the corresponding peptide, P7. P7-FLP is a full-length macrocyclic peptide. P7-RiP is a reinitiated peptide lacking ClAcXaa. The mRNA and peptide sequences for flag are GAC-UAC-AAG-GAC-GAC-GAC-GAC-AAG and Asp-Tyr-Lys-Asp-Asp-Asp-Asp-Lys. (**B, C**) Structures of ClAcPro and ClAc^3^Abz introduced at the peptide N-terminus. (**D, E**) MALDI-TOF MS of translated peptides. Translation was carried out using tRNA^iniP^_CUU_. Calculated (calc.) and observed (obs.) *m/z* values of [M + H]^+^ are indicated. (**F, G**) Chemical structures of macrocyclic P7-FLP bearing AcPro and Ac^3^Abz.

For macrocyclization of peptides, *N*-chloroacetylated amino acids were used instead of *N*-acetylated ones. Here, *N*-chloroacetyl-l-Pro (ClAcPro) and *N*-chloroacetyl-^3^Abz (ClAc^3^Abz) were precharged on tRNA^iniP^_CUU_ and introduced at the N-terminus of a model peptide, P7 (Figure 6A–C). Translation was carried out in the presence of 15 μM IF3, 1 μM EF-G, 10 μM EF-P, 2.5 μM RRF and 200 μM aminoacyl-tRNA^iniP^_CUU_. The *N*-chloroacetyl group spontaneously reacted with the sulfhydryl group of a downstream Cys to form an irreducible thioether bond, resulting in macrocyclization of peptide (42). MALDI-TOF MS of the translation products showed that the desired macrocyclic peptides were cleanly expressed without truncation (Figure 6D–G, P7-FLP-ClAcPro and P7-FLP-ClAc^3^Abz).

## DISCUSSION

Here, we have demonstrated that EF-P is able to enhance incorporation of diverse N-terminal substrates. We previously reported that EF-P recognizes the specific D-arm motif of tRNA^Pro^ isoacceptors comprised of a 9-nt D-loop closed with a 4-bp D-stem, where high GC content of D-stem is required for efficient recognition (35). The D-arm of tRNA^fMet2^ and tRNA^Pro1^ are almost identical except for one base pair at position 11 and 24 of the D-stem, where an A/U base pair is found in tRNA^fMet2^ and a C/G pair in tRNA^Pro1^. Therefore, it is reasonable that substitution of the A/U pair of tRNA^iniG1^, whose D-arm is derived from tRNA^fMet2^, with a C/G pair resulted in further improvement of the P1-FLP level and P1-FLP% in the presence of EF-P. The pyrimidine 11/purine 24 pair is unique and widely conserved in prokaryotic initiator tRNAs (Figure 1C) (43,44); However, it was reported that a mutant initiator tRNA bearing C/G pair at this position was quite active in protein synthesis *in vivo* (45). Similarly, the C/G mutation in our engineered initiator tRNAs was tolerated for AcPro incorporation.

By using the engineered initiator tRNA, tRNA^iniP^, under the optimized translation conditions, we succeeded in efficient incorporation of AcPro at the N-terminus with 1000-fold improvement of expression level compared to that of our conventional conditions using tRNA^iniWT^. Moreover, N-terminal drop-off-reinitiation event was completely suppressed under these conditions. The N-terminal Pro residue is an attractive building block of bioactive foldamer peptides owing to its constrained cyclic structure that contributes to stabilization of turn and helical conformations (1,2). d-Amino, β-amino and γ-amino acids are also useful building blocks of bioactive peptides that can be introduced by our methodology. We succeeded in introducing Ac^D^Trp, Ac^D^Tyr, Ac^β^Phg and Ac^3^Abz into model peptides as their representatives. We can expect their unique and strong folding propensities, such as turn/helix inducing abilities (3–12). Macrocyclization is also a powerful approach to construct constrained geometries of peptides. We showed that tRNA^iniP^ is applicable to introduction of *N*-chloroacetylated substrates, ClAcPro and ClAc^3^Abz, at the N-terminus for macrocyclization of peptides (Figure 6). We can expect high binding affinity and inhibitory activity against target molecules, improved membrane permeability, and proteolytic stability for these foldamer peptides (13–17). Peptides consisting of only l-α-amino acids are rapidly degraded by peptidases in serum or in cells, which is often a critical disadvantage of peptide drugs. However, by introducing noncanonical amino acids at the N-terminus, their proteolytic stability against exopeptidases drastically improves (46,47). In addition, their *N*-acetylation also increases proteolytic stability (48). The advantage of ribosomally synthesizing such exotic peptides is that, by translating mRNAs with random sequences, we can easily prepare random peptide libraries, which can be applied to display-based screening methodologies, such as the RaPID (Random nonstandard Peptides Integrated Discovery) system (40). RaPID screening of novel bioactive peptides bearing exotic amino acids at the N-terminus would be performed in our future studies.

## Supplementary Material

gkad496_Supplemental_Files

## Data Availability

The data underlying this article are available in the article and in its online supplementary material.
