# Supplementary material for: Translation initiation with exotic amino acids using EF-P-responsive artificial initiator tRNA
Source: Nucleic Acids Res. 2023 Jun 19;51(15):8169–80. doi: 10.1093/nar/gkad496 (PMC10450175; doi:10.1093/nar/gkad496)
Supplement: gkad496_Supplemental_Files [file gkad496_supplemental_files.zip › 230215iniP+caption_supplementary.pdf]

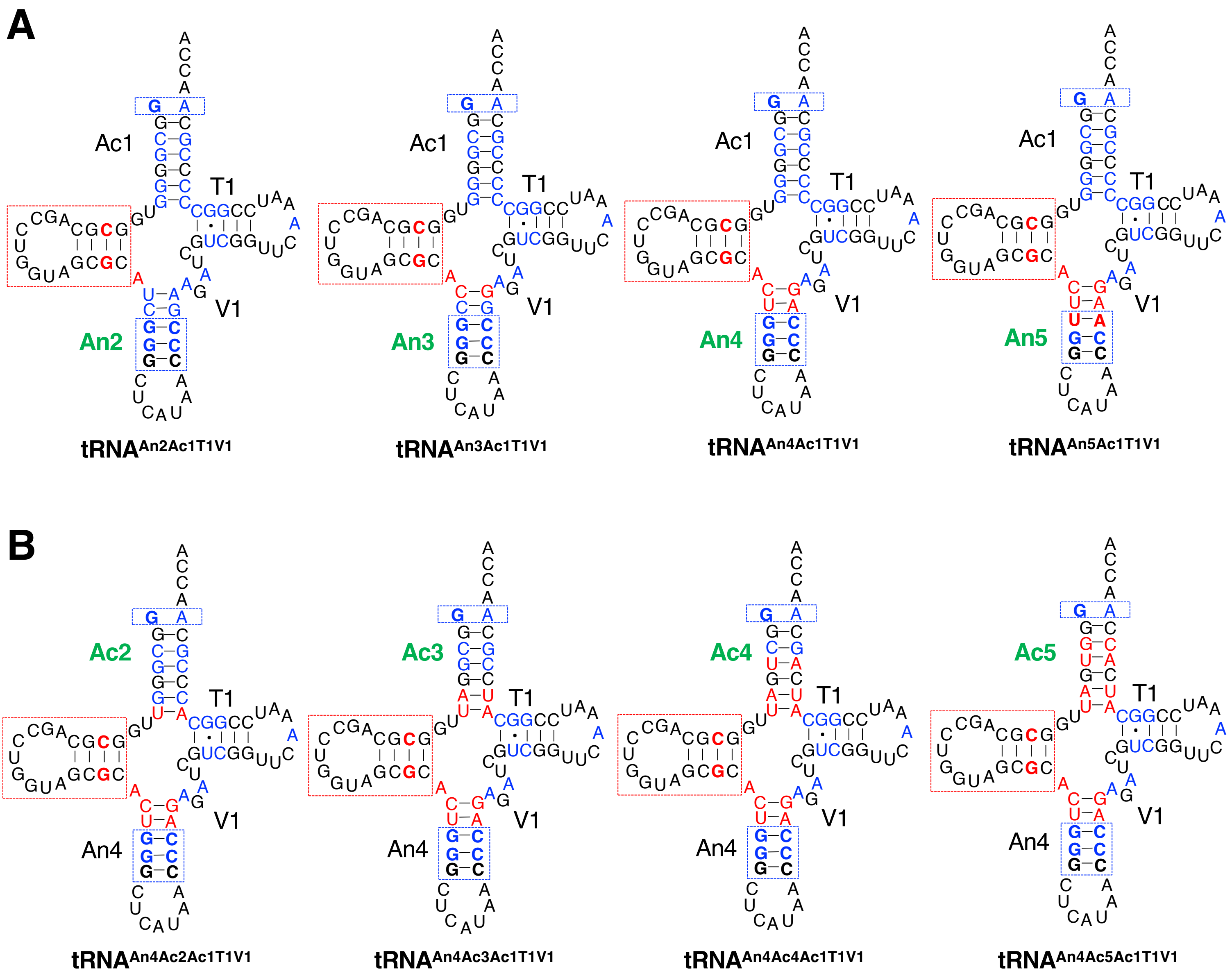

Supplementary Figure S1. Secondary structures of tRNA^{ini} variants. (A) anticodon stem variants. **(B)** acceptor stem variants. **(C)** T-stem/variable loop variants.

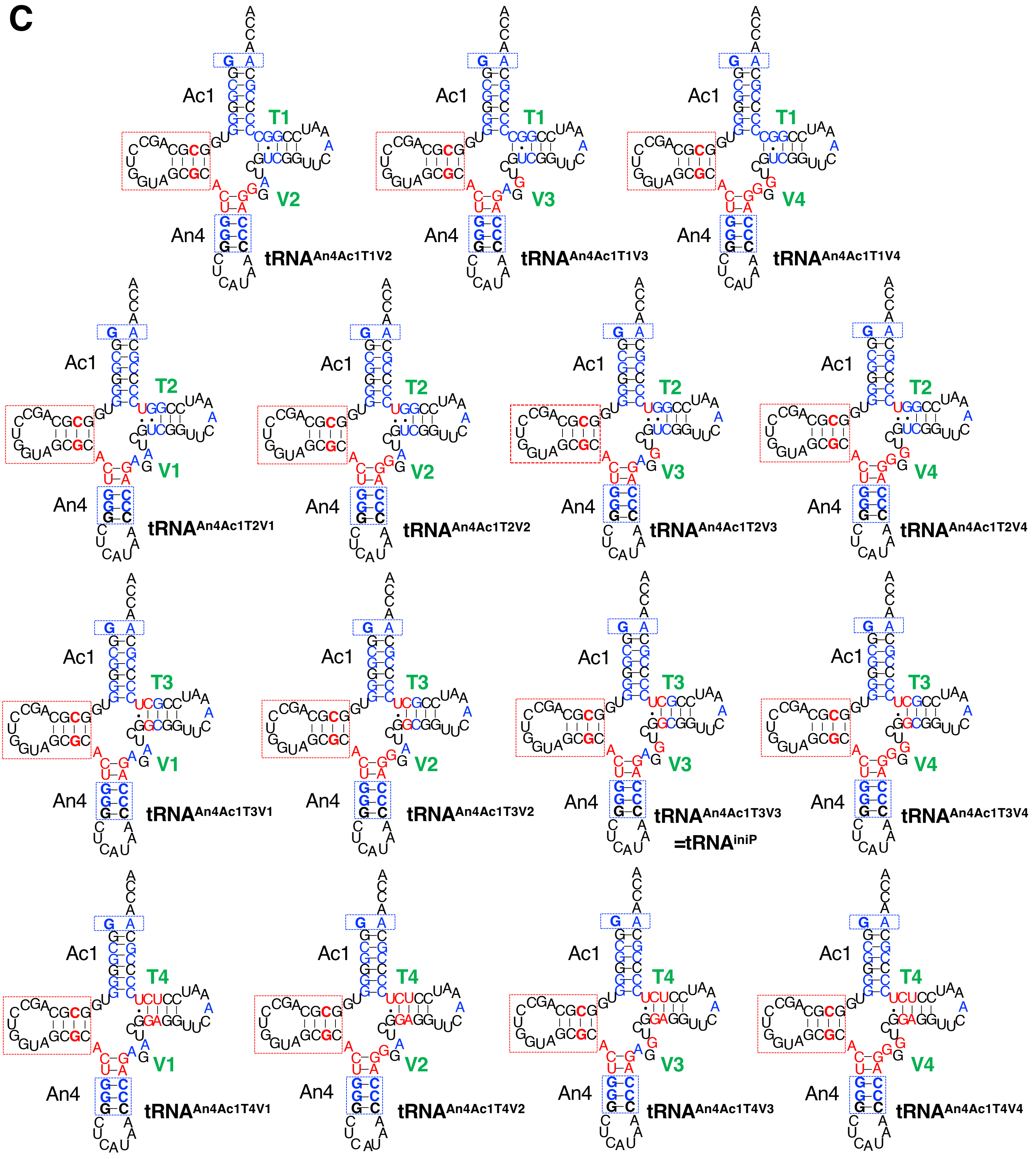

Supplementary Figure S1 continued.

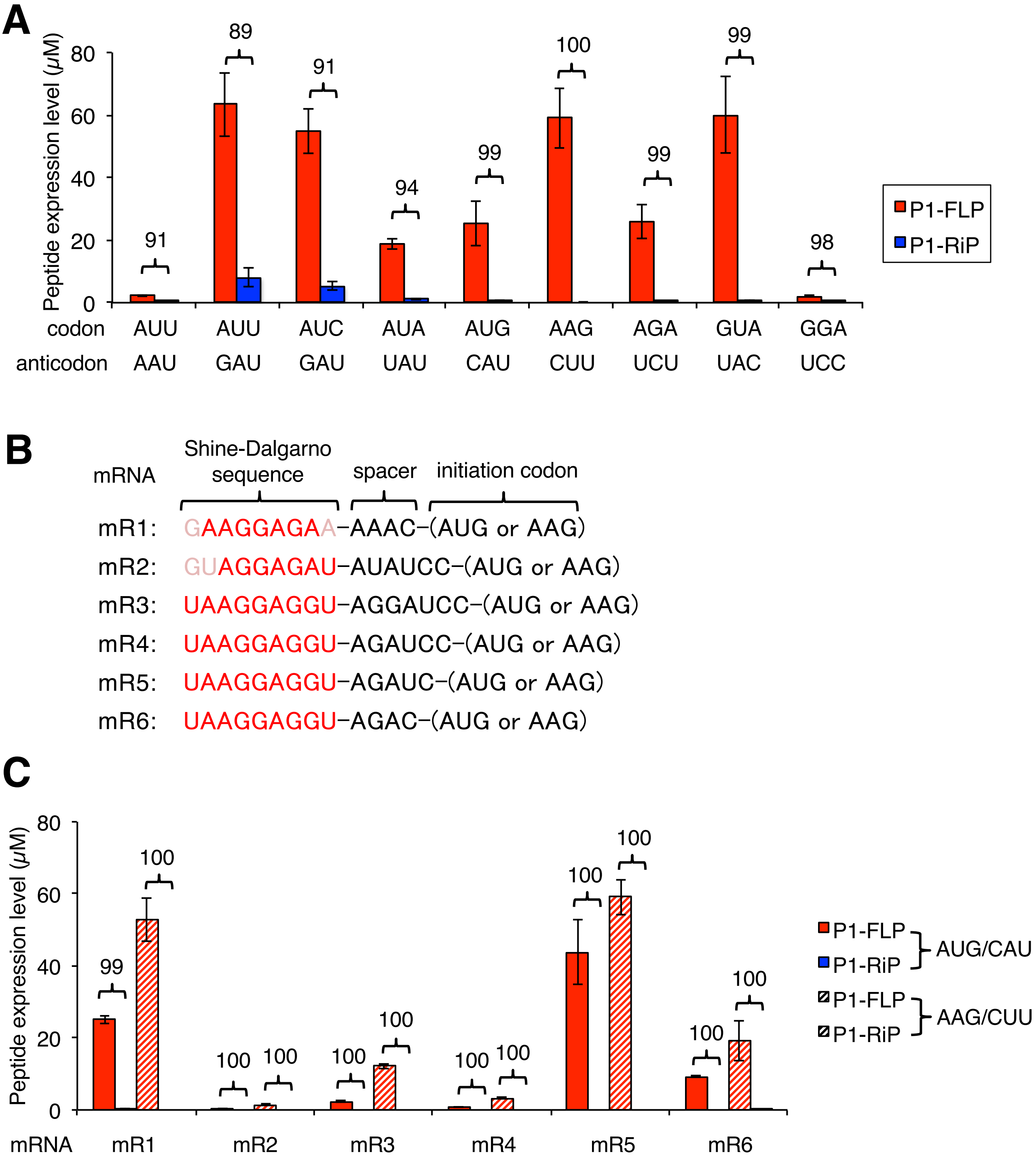

Supplementary Figure S2. Optimization of codon-anticodon combinations and Shine-Dalgarno sequence for incorporation of AcPro at the N-terminus. (A) Expression levels of P1-FLP and P1-RiP translated from mR1, where the initiation codon used for AcPro incorporation was changed as indicated at the bottom. 15 μM IF3, 1 μM EF-G, 2.5 μM RRF, 10 μM EF-P, and 160 μM AcPro-tRNA^{iniP} were used. The anticodon of tRNA^{iniP} was also changed accordingly as indicated at the bottom. Numbers above the bars show P1-FLP%. n = 3. Error bars, S.D. **(B)** Sequences of mRNAs, mR1−mR6. Shine-Dalgarno (SD) sequence and the spacer between SD and the initiation codon were optimized. Nucleotides that are complementary to the anti-SD of 16S rRNA are indicated by red, whereas pink indicates non-complementary ones. AUG or AAG was used as the initiation codon. **(C)** Expression levels of P1-FLP and P1-RiP translated from mR1−mR6. 15 μM IF3, 1 μM EF-G, 2.5 μM RRF, 10 μM EF-P, and 160 μM AcPro-tRNA^{iniP} were used. Numbers above the bars show P1-FLP%. n = 3. Error bars, S.D.
